# Commentary: Infusion line contamination in preterm neonates: impact of infusion line design, length, and use duration: the multicenter ChronoBIOline study

**DOI:** 10.3389/fmicb.2025.1581152

**Published:** 2025-07-14

**Authors:** Jean-Charles Picaud, Stéphane Hays, Cédric Dananché

**Affiliations:** ^1^Service de Néonatologie, Hôpital de la Croix-Rousse, Hospices Civils de Lyon, Lyon, France; ^2^Laboratoire CarMen, Faculté de Médecine Lyon-Sud Charles Mérieux, Université Claude Bernard Lyon 1, Pierre-Bénite, France; ^3^Département d'Hygiène Hospitalière, Épidémiologie et Prévention, Hôpital de la Croix-Rousse, Hospices Civils de Lyon, Lyon, France; ^4^CIRI, Centre International de Recherche en Infectiologie, INSERM, U1111, Université Claude Bernard Lyon 1, CNRS, UMR5308, ENS de Lyon, Lyon, France

**Keywords:** infection, parenteral nutrition, infusion, central venous line, catheter

## Introduction

In the ChronoBIOline study, bacterial contamination was detected in 22.2% of 108 infusion sets with 28 different designs, and the conclusion was that multiline systems were at high risk of contamination (Dos Santos et al., 2025). Nevertheless, several concerns over the study remain that prevent the formation of firm conclusions.

### Contamination and infection

The first methodological concern is the intermediate outcome, as colonization of the catheter is not always associated with bloodstream infection (Narendran et al., 1996; Cronin et al., 1990). In the ChronoBIOline study, the authors collected lines when they were changed or removed, regardless of the presence of an infection. As data about infections was not collected, the results preclude the inference that colonized catheters will lead to an infection, which the authors noted (Dos Santos et al., 2025). Moreover, published studies investigating the association between contamination of infusion sets and infection in neonatal intensive care units (NICU) remain poor and report conflicting results (Narendran et al., 1996; Cronin et al., 1990). Furthermore, after implementation of a multiline multilumen infusion set in a tertiary care neonatal unit in 2020, we found a drastic reduction in the rate of central venous line-associated bloodstream infection (CLABSI; −88%) (Picaud et al., 2024).

### Minor and major contamination

The second concern is over the definition of major contamination (≥50 colony-forming units, CFU) (Dos Santos et al., 2025), which is not supported by any reference reporting that the risk of infection is significant above this. Methodologically, the use of this threshold should have been justified, for example by an additional risk of infection if infusion lines are colonized above this number. Accordingly, data about the risk related to major contamination cannot be interpreted.

### Micro-organism recovery

Infusion sets were removed and sent to the coordinating center, which might have been a source of contamination, and information about the time needed to transfer sets was not reported (Dos Santos et al., 2025). The method used for sample processing was derived from a procedure originally used to detect microbial biofilm in flexible endoscope channels (Noubam-Tchatat et al., 2023), but the authors did not validate this for all types of infusion sets tested; notably, no multiline multilumen system was tested, although the authors deliver a key message concerning these systems (Dos Santos et al., 2025).

### Identified micro-organisms

The micro-organisms identified in contaminated infusion lines seem to reflect cutaneous or common environmental flora. In France, coagulase-negative *Staphylococcus* spp. represent the vast majority of microorganisms identified in cases of CLABSI (Picaud et al., 2024; Surveillance Des Cathéters Centraux En Néonatologie, 2019). In the ChronoBIOline study, the authors reported that nearly half of infusion lines (10/24, 41.7%) were contaminated with 10 different species of non-cereus *Bacillus* (Dos Santos et al., 2025), a well-known environmental microorganism (Hosein et al., 2013; Shimono et al., 2012). Most contamination reports (15/24, 62.5%) appear to be ≤ 5 CFUs of cutaneous or environmental flora, suggesting contamination that could have originated from manipulations of infusion sets at removal, during transport, or when sampling for micro-organism recovery (Dos Santos et al., 2025).

### Heterogeneity of the infusion systems analyzed

Infusion sets were collected in ~20% of French NICUs without specific selection, which does not ensure representativeness of the systems used nationally. In addition, the sets were 10–180 cm in length, with 1 to 6 “parts”, with or without filters, open or closed, and single or multiline. As a consequence, only a very small number of each system was analyzed, which does not allow reliable comparisons. Furthermore, there was heterogeneity among the 21 “multiline” systems, as 18 were Edelvaiss^®^ and three were other systems. However, the former stands out from the others because it is a closed multiline multilumen system which is fully assembled by the manufacturer prior to sterilization and end-user packaging (Picaud et al., 2024; Martelin et al., 2024; Foinard et al., 2013). Other systems are open systems, assembled at the bedside from several components sourced from one or multiple manufacturers. Edelvaiss^®^, which was part of the infusion line tested, is a new infusion system composed of eight lumens within the same tube to reduce physical incompatibility and drug delivery disturbances during simultaneous administration of drugs in neonates (Shimono et al., 2012). As it is a closed system, it requires fewer manipulations (fewer connections/disconnections and a purging system), and the long line allows this to be done at a distance from the patient; both preserve the infant's autonomic stability and sleep and help to reduce the risk of CLABSI (Picaud et al., 2024). Although the ChronoBIOline study provides useful data, a follow-up study using standardized infusion set systems is needed for more precise comparisons.

### Duration of infusion systems and risk of contamination

As the duration of use of the infusion systems is considered a risk factor of bacterial contamination, it is recommended that the infusion set is removed before seven days (World Health Organization, 2024; PICC, 2013). This was based on five studies, none of which included neonates and reported only the use of legacy infusion sets, which are open systems composed of a single lumen with multiple stopcocks and connectors (Guidelines for the Prevention of Intravascular Catheter-Related Infections, 2011); the Edelvaiss^®^ system, however, was specifically designed for neonates and is certified for use up to 21 days. Furthermore, although there is a significant difference in the frequency of contamination over time, stratified according to whether the set was a multi- or single-line system, a significant difference was found only during the first 4 days (Dos Santos et al., 2025).

## Discussion

Although the ChronoBIOline study provides valuable insights into contamination risks, its design (i.e. observational, descriptive, and based on an intermediate outcome) is not able to assess the effectiveness and safety of multiline multilumen infusion lines. The authors present raw results of infusion set colonization, but a center effect might exist (as noted in the paper), as participating NICUs use different infusion sets and different infection prevention measures (Dos Santos et al., 2025). Furthermore, the adherence to these measures, which is a major risk factor of CLABSI, might vary according to the NICU (Zachariah et al., 2014). Finally, the implementation of a multi-infusion device requires a strict training program for nurses, with regular assessment of practices (Picaud et al., 2024). The absence of these data (Dos Santos et al., 2025) limits the interpretation of the results. The authors concluded that “Overall, our data suggest that the use of the multiline systems could promote CLABSIs in preterm neonates” and that their results support current guidelines i.e. “simpler infusion lines” with a maximum duration of 7 days (Dos Santos et al., 2025), but neither the study design nor the data analysis support this conclusion. Harmonized outcome definitions and more robust designs are needed. In addition, clinical benefits conferred by the use of multiline multilumen devices in preterm infants, including drug administration and monitoring, must be considered in their evaluation. Further well-designed, prospective randomized studies are needed in order to address the aim of the ChronoBIOline study.
